# Molecular profiling of prostate cancer derived exosomes may reveal a predictive signature for response to docetaxel

**DOI:** 10.18632/oncotarget.3226

**Published:** 2015-03-12

**Authors:** Pedram Kharaziha, Dimitris Chioureas, Dorothea Rutishauser, George Baltatzis, Lena Lennartsson, Pedro Fonseca, Alireza Azimi, Kjell Hultenby, Roman Zubarev, Anders Ullén, Jeffrey Yachnin, Sten Nilsson, Theocharis Panaretakis

**Affiliations:** ^1^ Department of Oncology-Pathology, Karolinska Institutet and University Hospital, Stockholm, Sweden; ^2^ Department of Medical Biochemistry and Biophysics, Karolinska Institutet and University Hospital, Stockholm, Sweden; ^3^ Science for Life Laboratory, Stockholm, Sweden; ^4^ Department of Medicine, School of Health Sciences, University of Athens, Athens, Greece; ^5^ Department of Laboratory Medicine, Karolinska Institutet and University Hospital, Huddinge, Sweden

**Keywords:** Prostate cancer, exosomes, docetaxel, resistance, biomarkers

## Abstract

Docetaxel is a cornerstone treatment for metastatic, castration resistant prostate cancer (CRPC) which remains a leading cause of cancer-related deaths, worldwide. The clinical usage of docetaxel has resulted in modest gains in survival, primarily due to the development of resistance. There are currently no clinical biomarkers available that predict whether a CRPC patient will respond or acquire resistance to this therapy. Comparative proteomics analysis of exosomes secreted from DU145 prostate cancer cells that are sensitive (DU145 Tax-Sen) or have acquired resistance (DU145 Tax-Res) to docetaxel, demonstrated significant differences in the amount of exosomes secreted and in their molecular composition. A panel of proteins was identified by proteomics to be differentially enriched in DU145 Tax-Res compared to DU145 Tax-Sen exosomes and was validated by western blotting. Importantly, we identified MDR-1, MDR-3, Endophilin-A2 and PABP4 that were enriched only in DU145 Tax-Res exosomes. We validated the presence of these proteins in the serum of a small cohort of patients. DU145 cells that have uptaken DU145 Tax-Res exosomes show properties of increased matrix degradation. In summary, exosomes derived from DU145 Tax-Res cells may be a valuable source of biomarkers for response to therapy.

## INTRODUCTION

Prostate cancer (PCa) is the most frequent malignancy in men in western countries and it is the second cause of cancer mortality. Localized prostate cancer is treated by androgen deprivation therapy, radiotherapy and/or surgery but frequently patients develop resistance and progress to castration-resistant prostate cancer (CRPC) [1]. Docetaxel is currently the first line therapy standard for patients with metastatic CRPC. Unfortunately, the average median survival of CRPC patients is modestly increased with docetaxel compared to mitoxantrone by about 3 months [2].

Docetaxel binds to β-tubulin and prevents disassembly of the microtubule network which can be detrimental for the cells since it leads to the stabilization of the mitotic spindle during the G2-M phase of the cell cycle leading to cell death by mitotic catastrophy. Docetaxel acts also by inhibiting anti-apoptotic Bcl2 family members, such as Bcl-2, which lowers the apoptotic threshold and allows for stressed cells to undergo apoptosis [3].

There are several, well-described mechanisms of acquisition of resistance to docetaxel either due to intrinsic prostate cancer biology or generic drug resistance mechanisms [4]. The former include (i) sustained androgen receptor (AR) signaling due to overexpression of AR, coactivator of the AR or androgen production inside the prostate tumor; (ii) activation of tyrosine kinase receptor IGFR-1, EGFR, VEGFR and downstream signal transduction pathways like PI3K/AKT and Ras/Raf/MEK/ERK pathways; (iii) aberrant angiogenesis, (iv) stroma-derived cytokines and growth factors which promote cell growth and resistance to chemotherapy. The latter includes generic resistance mechanisms include (i) impaired drug distribution; (ii) survival cues driven by the tumor microenvironment; and (iii) overexpression of multi-drug resistance proteins (e.g. the ATP-binding cassette (ABC) transporters MDR-1, MDR-2 and MRP1 (ABCC1)) which excrete docetaxel into extracellular fluid and decrease the lethal, intracellular concentration of the drug [4].

There are currently very few biomarkers that would predict whether patients treated with docetaxel will respond and/or acquire primary/secondary resistance. The golden standard, prostate specific antigen (PSA) is commonly used but has its limitations that include, among others, lack of PCa specificity and lack of indication of early therapeutic response [5, 6]. Thus, there is an urgent and unmet need for the discovery of novel prognostic biomarkers and especially predictive biomarkers that will stratify patients based on the most efficient therapeutic strategies [5].

Cells secrete a wide variety of extracellular vesicles (EVs) under both physiological and pathological conditions, with the most studied type of EVs being the exosomes [5]. Exosomes are endosome-derived vesicles with a diameter between 50 and 150 nm and they float at a density between 1.13 and 1.19 g/ml on a linear sucrose gradient. Exosomes have been shown to play a wide variety of physiological roles, e.g. immune response modulation, presentation of antigens to immune cells, intercellular communication through transfer of DNA, RNA and proteins [5]. It is becoming more and more evident that cancer cells secrete high levels of EVs into the blood circulation that can be isolated and characterized [7, 8]. The multiplex molecular composition of these EVs that may be considered as PCa and CRPC cell fingerprints has put them into the center of the biomarker research since they may allow for detection of malignancy by non-invasive means [5]. In fact several studies have performed proteomic and transcriptomic arrays in the EVs secreted by metastatic and non-metastatic cells and have identified several proteins and miRNAs that may be used as prognostic biomarkers for progression of indolent disease to castration resistant prostate cancer as well as to metastatic CRPC [9–15]. However, none of the studies published so far has examined the EVs as a source of predictive biomarkers for response to therapy.

In this study we present our data obtained from the comparative proteomics analysis on exosomes isolated from DU145 Tax-Sen and DU145 Tax-Res cells. This analysis revealed that the molecular composition of these EVs is significantly different and it includes several proteins that may be used as predictive biomarkers of therapeutic response or resistance.

## RESULTS AND DISCUSSION

### Characterisation of extracellular vesicles secreted from docetaxel sensitive and resistant DU145 cells

**Figure 1:**
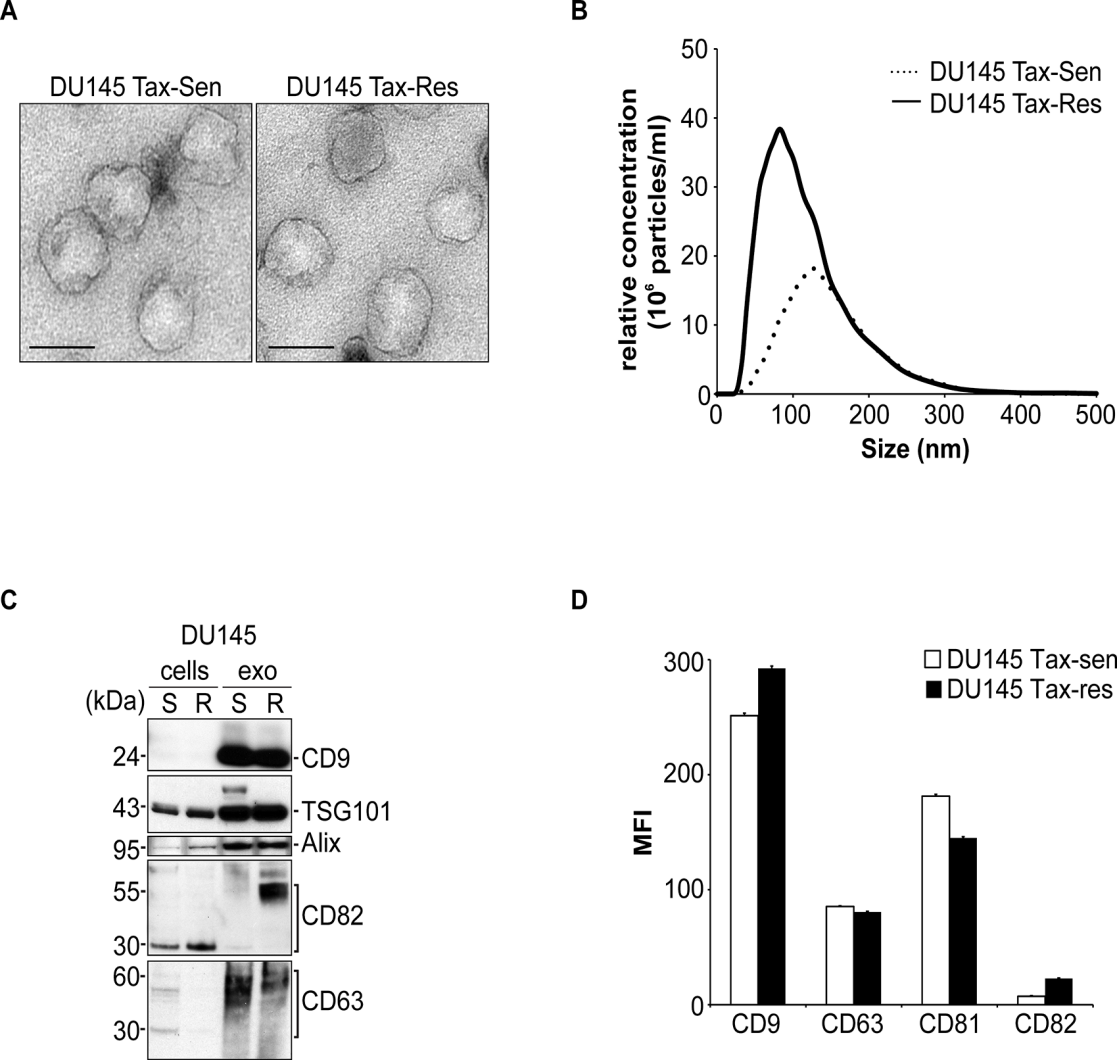
Characterization of extracellular vesicles secreted from docetaxel sensitive and resistant DU145 cells **(A)** Exosomes isolated from DU145 Tax-Sen and DU145 Tax-Res cells were visualized by transmission electron microscopy (bar size: 100 nm); **(B)** Nanoparticle tracking analysis on an LM10 Nanosight demonstrating a mean size of 100 nm for DU145 Tax-Res and 120 nm for DU145 Tax-Sen exosomes. The size distribution and relative concentration were calculated by the Nanosight software (*n* = 3); **(C)** Western blot analysis of 10 μg lysates from DU145 Tax-Sen and DU145 Tax-Res cells and exosomes and probed for the indicated proteins (*n* = 2); **(D)** Flow cytometric analysis of the mean fluorescence intensity (MFI) for a panel of exosomal markers CD9, CD63, CD81 and CD82. Data is presented as means of triplicate experiments.

We have generated DU145 cells that are stably resistant to 500 ng/ml docetaxel and extracellular vesicles (EV) were isolated from DU145 sensitive (DU145 Tax-Sen) and resistant (DU145 Tax-Res) cell supernatants. The resistance of the established cell line to docetaxel was examined by measuring cell death in response to this anti-cancer drug for 24 and 48 hours (Supplementary Figure 1). Transmission electron microscopy revealed similar morphological characteristics between the DU145 Tax-Sen and DU145 Tax-Res EVs with a homogeneous structure and a median diameter of about 100 nm (Figure 1A). Nanoparticle tracking analysis (NTA) of the isolated exosomes revealed that the DU145 Tax-Res cells secrete about 2 to 3 times higher amounts of EVs compared to the DU145 Tax-Sen ones (Figure 1B). It is intriguing that the resistant cells secrete higher amount of vesicles compared to the sensitive DU145 cells in response to an agent that targets the microtubule which have been shown to regulate vesicular trafficking and are implicated in exosome secretion [16, 17]. It is tempting to speculate that this is a cellular defense mechanism that has been activated during the phase of acquisition of resistance. The potential, re-programmed mechanisms of exosome secretion that are activated in DU145 Tax-Res are now a subject of intense investigation in our laboratory. Immunoblot comparative analysis for generic EV markers showed that the DU145 Tax-Sen and the DU145 Tax-Res EVs are enriched with equal amounts of TSG101, CD9 and Alix (Figure 1C). Differences were observed for CD82 which seems to migrate slower in DU145 Tax-Res derived exosomes and may be indicative of the glycosylation status of CD82 (Figure 1C). Interestingly, it has been previously shown that CD82 can be heavily glycosylated and demonstrates a molecular weight between 50–60 kDa [6]. Further characterization of the EVs by flow cytometry for these EV markers did not reveal any significant differences between the DU145 Tax-Sen and Tax-Res cell derived exosomes (Figure 1D). Collectively, the obtained data indicate that the morphological and the marker profile of the EVs secreted from DU145 Tax-Sen and Tax-Res cells is relatively similar with the major difference identified on the amount of EVs secreted and the levels of CD82 detected.

### Comparative biochemical characterization of exosomes isolated from docetaxel sensitive and resistant DU145 cells

**Figure 2:**
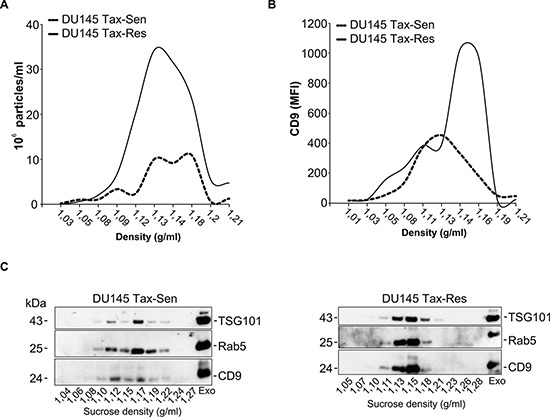
Comparative biochemical characterization of exosomes isolated from docetaxel sensitive and resistant DU145 cells **(A)** Nanoparticle tracking analysis of sucrose gradient fractions with the indicated sucrose density. The size distribution and relative concentration were calculated by the Nanosight software (*n* = 3); **(B)** Flow cytometric analysis for the mean fluorescence intensity (MFI) of CD9-APC in the sucrose gradient fractions (*n* = 2); **(C)** Western blot analysis of the sucrose gradient fractions from DU145 Tax-Sen and DU145 Tax-Res exosomes, probed for Rab 5, TSG101 and CD9, (*n* = 2).

The EVs were further characterized with regard to their density distribution on a linear sucrose gradient. Typically exosomes float between 1.13 and 1.19 g/ml on a linear sucrose gradient. In agreement with the literature, nanoparticle tracking analysis (NTA) demonstrated that the majority of the isolated EVs displayed a buoyant density of 1.12 to 1.19 g/ml, characteristic for exosomes (Figure 2A) [18, 19]. Importantly, NTA further confirmed the significant difference in the amount of secreted particles from DU145 Tax-Sen and DU145 Tax-Res cells (Figure 2A). Flow cytometric analysis of the sucrose gradient fractions for CD9-APC revealed the differential enrichment of this exosomal marker in the DU145 Tax-Sen and DU145 Tax-Res derived exosomes (Figure 2B and Supplementary Figure 2). We examined the presence of classical EV markers, i.e. TSG101, Rab5 and CD9 in the sucrose gradient fractions by western blotting (Figure 2C). It seems that the DU145 Tax-Sen exosomes that are enriched in CD9, Rab5 and TSG101, show a broader distribution in the linear gradient, floating in densities between 1.12 and 1.19 g/ml and the DU145 Tax-Res derived exosomes at a more narrow sucrose density between 1.13 to 1.18 g/ml. These data further illustrate that the exosomes secreted by the DU145 Tax-Sen and DU145 Tax-Res cells are different not only in amounts but also in the physical characteristics of the secreted exosomes.

### Proteomics profiling of exosomes isolated from docetaxel sensitive and resistant DU145 cells

**Figure 3:**
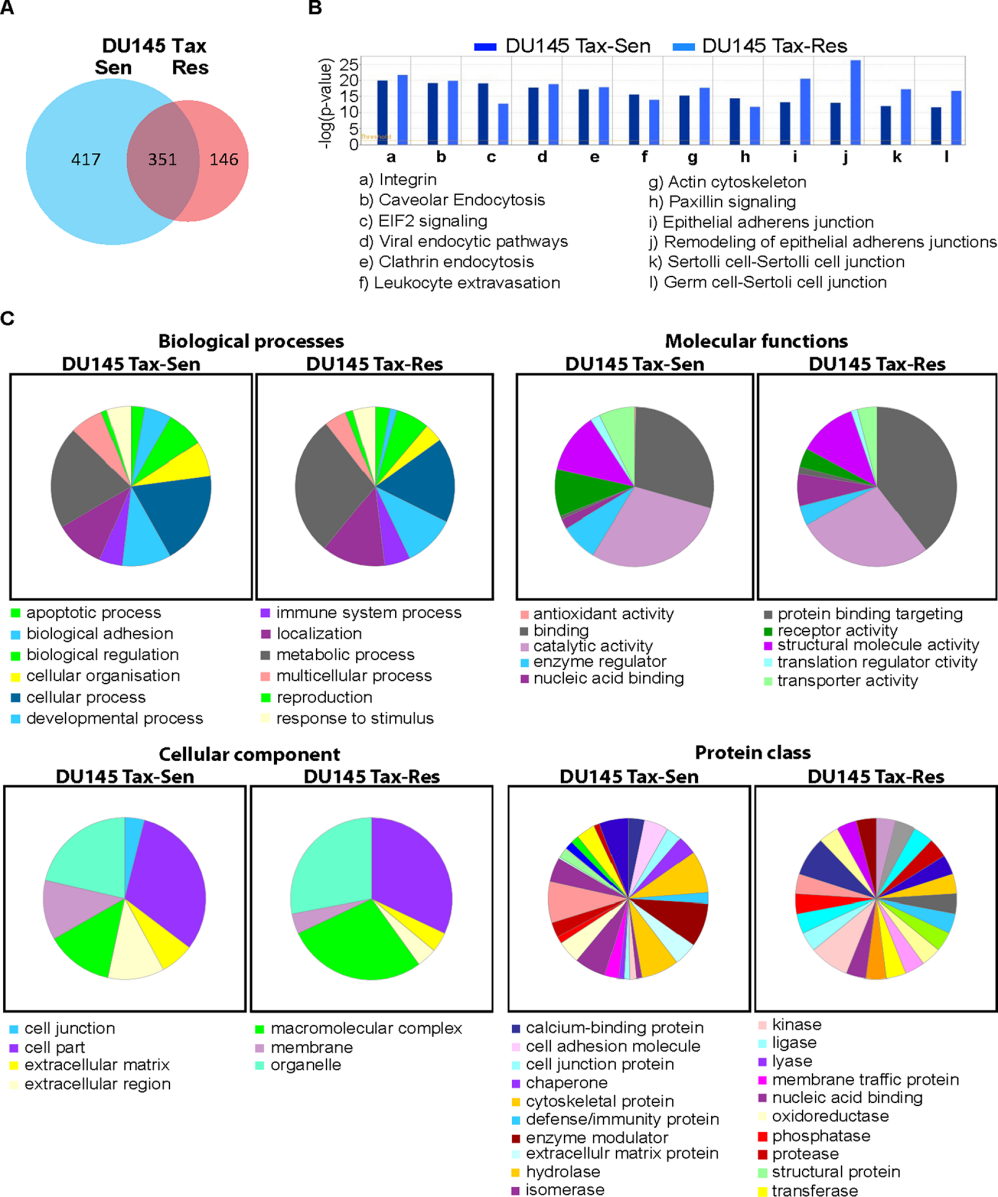
Bioinformatics analysis of the DU145 Tax-Sen and DU145 Tax-Res exosomal proteome **(A)** Venn Diagram constructed from the unique and shared proteins identified in DU145 Tax-Sen and DU145 Tax-Res exosomes; **(B)** Ingenuity pathway analysis of the signaling cascades identified in the proteomics analysis; **(C)** Pie charts from the classification analysis (PANTHER 9.0) of the biological processes, molecular functions, cellular components and protein classes.

Proteomics analysis of DU145 Tax-Sen and DU145 Tax-Res exosomes, isolated from the supernatants of the corresponding cell lines, by nLC-MS/MS analyses identified 914 proteins with at least one peptide with an FDR ≤ 1% (Supplementary Table 1). Of these, 351 proteins were common in both, 417 were unique for DU145 Tax-Sen and 146 proteins were unique for DU145 Tax-Res exosomes (Figure 3A). Bioinformatic analysis using the Ingenuity software revealed pathways in which the identified proteins are known to be part of. In the majority of the top 12 identified pathways, the observed differences were not significant (Figure 3B). The most prominent differences were found in the eIF2 signaling cascade for DU145 Tax-Sen exosomes, the epithelial adherence junctions and the remodeling of adherence junctions for DU145 Tax-Res exosomes (Figure 3B). Further classification analysis using PANTHER™ revealed additional differences in the proteomes between DU145 Tax-Sen and DU145 Tax-Res exosomes (Figure 3C). Interestingly, proteins associated with metabolic activity are enriched in exosomes isolated from DU145 Tax-Res exosomes whereas those involved in biological adhesion and cellular organization are found mostly in DU145 Tax-Sen derived exosomes. With regard to molecular functions, DU145 Tax-Sen exosomal proteins may play a role in enzyme regulator and receptor activity whereas DU145 Tax-Res exosomal proteins may participate in nucleic acid binding. This is of particular interest, since it was recently shown that exosomes apart from RNA species also contain DNA [7]. Cellular component and protein class analysis further demonstrated that the exosomes derived from these two prostate cancer cell lines have subtle but distinct differences that may regulate their biological function of recipient cells.

**Figure 4:**
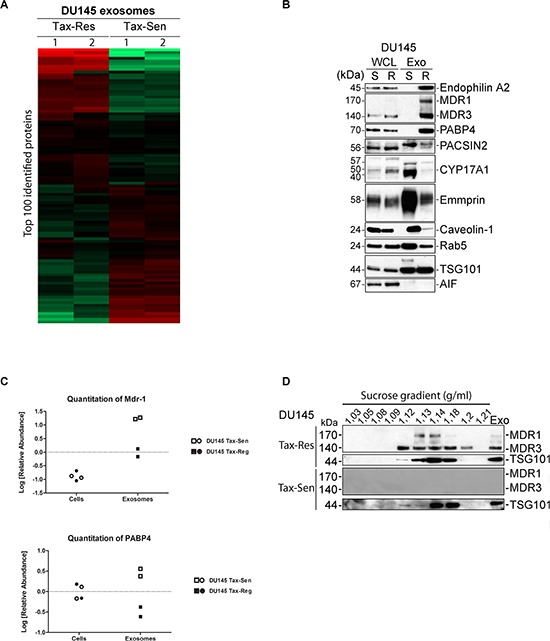
Proteomics profiling of exosomes isolated from docetaxel sensitive and resistant DU145 cells **(A)** Heat map of the hierarchical clustering of the 100 most abundant protein identified. The values were mean centered and log-transformed. The relative protein abundance is colored-coded with red corresponding to a relatively high abundance, green to a relatively low abundance, and black indicating indifferent abundance values. Each exosome sample was analyzed in duplicates; **(B)** Western blot analysis of 10 μg of DU145 Tax-Sen and DU145 Tax-Res exosomes, probed for the indicated proteins. Rab5 and TSG101 were used as exosomal markers and AIF as a quality control of the exosomal isolation (*n* = 2); **(C)** Quantification of the abundance values for MDR-1 and PABP4; **(D)** Western blot analysis of the sucrose gradient fractions from DU145 Tax-Sen and DU145 Tax-Res exosomes, probed for MDR-1/3 and TSG101 (*n* = 2).

Unsupervised hierarchical clustering of proteins identified in DU145 Tax-Sen and DU145 Tax-Res exosomes revealed a clear difference in abundance between sensitive and resistant cell lines as shown in the heat map of the top 100 most abundant proteins (Figure 4A). We then selected the proteins that show the biggest difference in abundance levels between the DU145 Tax-Sen and DU145 Tax-Res exosomes and validated their enrichment in DU145 Tax-Res exosomes by western blotting. We used Rab5 and TSG101 as exosomal markers and AIF as a quality control for our isolations (Figure 4B). AIF is an intra-mitochondrial protein that would only leak if the cells are dying or have compromised their mitochondrial membrane potential, an event that usually precedes cell death [20]. The most abundant protein found in DU145 Tax-Res exosomes compared to DU145 Tax-Sen was Endophilin-A2. This protein belongs to the BAR domain protein superfamily which is known to have a prominent role in clathrin-mediated endocytosis of synaptic vesicles [21]. We confirmed its presence in DU145 Tax-Res derived exosomes by western blotting (Figure 4B). Interestingly we could detect it only in DU145 Tax-Res exosome lysates with very little or non-detectable levels in DU145 Tax-Sen exosomes. Another highly abundant protein identified in the exosomes from DU145 Tax-Res was MDR-1 (Figure 4B). MDR-1 or p-glycoprotein (ABCB1) is a cell surface glycoprotein that mediates the ATP-dependent drug efflux pump and often promotes the development of chemoresistance to anticancer drugs. Even though the MDR-1/3 protein levels in the cells are similar, there is a very big difference in the protein levels found in the exosomes (Figure 4B and 4C). MDR-1 was previously detected by Corcoran *et al*., in their studies on the potential of docetaxel resistant cell derived exosomes to change the phenotype of recipient cells [22]. In addition to that study we could also detect MDR-3 in the exosomes isolated from DU145 Tax-Res cells. MDR-3 (ABCB4), another member of the multidrug resistance subfamily, has been shown to mediate the ATP-dependent export of organic anions and drugs from the cytoplasm. MDR-3 has also been shown to be a lipid translocase with specificity for phosphatidylcholine [23]. It is of particular interest that DU145 Tax-Res exosomes are enriched with one of the defense mechanisms that the cells possess to expel cytotoxic agents such as anti-cancer drugs. It was recently published that exosomes secreted from breast cancer cells overexpressing HER2 are capable of scavenging trastuzumab and thereby minimizing the available drug concentration that can reach and kill the breast cancer cells [24]. It is tempting to speculate that, in a similar manner to HER2 enriched exosomes, MDR-1/3 loaded exosomes can act as a systemic defense mechanism where they upload free docetaxel in the circulating exosomes. Alternatively, exosomes are used as a bulk docetaxel excretion mechanism, where multiple docetaxel molecules are pumped into exosomes in an MDR-1/3 dependent manner and then excreted from the cells. Whether any of the aforementioned scenarios are actual and whether the presence of MDR-1/3 in the exosomes is a cause or an effect of docetaxel resistance remains to be determined in our future investigations.

We subjected the isolated exosomes to a sucrose gradient and probed for MDR-1/3 as well as TSG101 as an exosomal control. We found a good correlation between the flotation of exosomes as judged by TSG101 that was detected in the fractions between 1.13 to 1.18 g/ml and the presence of MDR-1/3 that was more concentrated in fractions with sucrose density between 1.12 to 1.2 g/ml (Figure 4D). These data further confirmed the presence of MDR-1 and MDR-3 in our exosomes isolated from DU145 Tax-Res and the complete absence of this protein in exosomes isolated from DU145 Tax-Sen cells.

We went through the proteomics results and validated other proteins that were either highly abundant in exosomes or because they have been indicated in the literature to be promising biomarkers. PABP4, a poly(A)-binding protein at the 3-prime ends of most eukaryotic mRNAs [25], is another protein that is highly abundant in DU145 Tax-Res but not found in DU145 Tax-Sen exosomes according to the proteomic analysis (Figure 4B and 4C). The western blot data confirmed the proteomic analysis demonstrating the enrichment of PABP4 in the resistant cell derived exosomes. The presence of an mRNA binding protein is expected since it is well established that exosomes contain mRNA species in their cargo [26]. The enriched levels of PABP4 in the DU145 Tax-Res exosomes may reflect increased levels of mRNA in these exosomes and this interesting indication requires further investigation.

PACSIN2 is a lipid binding protein that has been shown to play a role in vesicular trafficking and was found in our proteomic study to be enriched in DU145 Tax-Res exosomes. However, validation by western blotting did not confirm this observation but rather showed decreased levels in DU145 Tax-Res exosomes. This disagreement between the proteomics data and the western blot analysis may reflect post-translational modifications that mask the epitopes recognized by the two different antibodies that we have used and prevent its detection.

Abiraterone acetate, an FDA approved drug, delivers a median 4-month survival benefit in docetaxel-refractory metastatic prostate cancer. [27]. It acts by inhibiting CYP17, an important enzyme in the synthesis of androgens and estrogens and thereby prevents the bioavailability of androgen receptor substrates [28, 29]. Since this chemical inhibitor depends on the presence of CYP17 we examined whether we could detect this enzyme in the exosomes secreted by DU145 Tax-Sen and DU145 Tax-Res derived exosomes. Interestingly, CYP17 could be detected in high amounts in the exosomes secreted by DU145 Tax-Sen cells but it was present in very low levels in the DU145 Tax-Res exosomes (Figure 4B).

Emmprin (CD147) is a cell surface multifunctional glycoprotein that has been shown to promote PCa metastasis and resistance to therapy by modifying the tumor microenvironment and regulating multidrug resistance [30]. Our proteomic analysis showed that Emmprin was lowered in DU145 Tax-Res derived exosomes, a finding that was confirmed by western blot (Figure 4B).

Caveolin-1 is a major structural component of caveolae, facilitating the cellular processes including molecular transport and cell adhesion [31]. Its expression levels correlated well with prostate cancer metastasis leading to its identification as a novel prognostic marker [32]. Even though it has significant prognostic value for metastatic CRPC, little is known about its predictive value for resistance to therapy. We evaluated the total protein levels of Caveolin-1 both in the cells and in the exosomes and found that it is abundantly expressed in the whole cell lysates and also present in the lysate of exosomes derived from DU145 Tax-Sen cells (Figure 4B). In the western blot analysis we found very little Caveolin-1 in the exosomes isolated from the DU145 Tax-Res. The significance of this finding for the metastatic propensity of the cells is not clear.

### Validation of selected proteins in castration resistant prostate cancer patient samples

**Figure 5:**
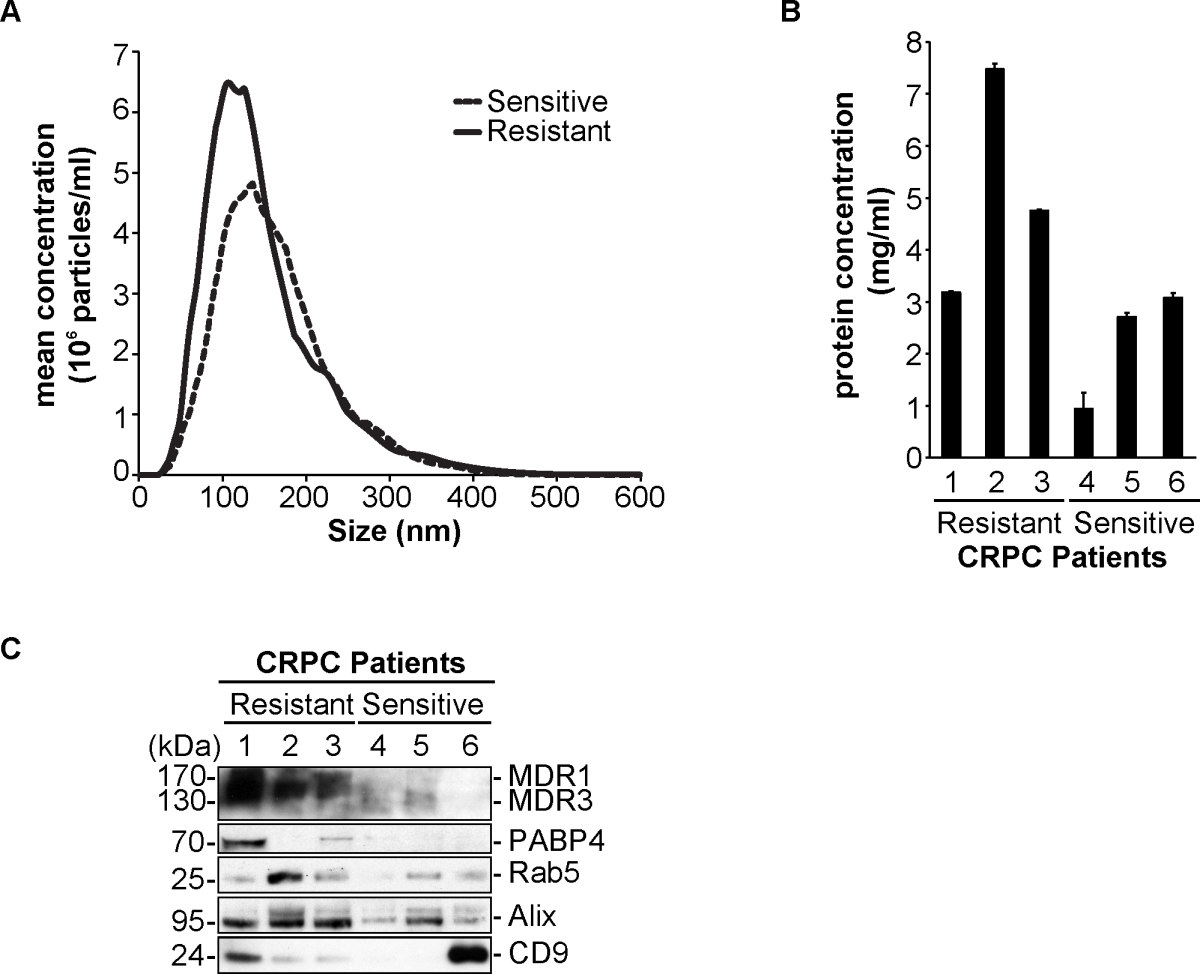
Validation of MDR-1/3 and PABP4 in the serum of castration resistant prostate cancer patients **(A)** Nanoparticle tracking analysis of the mean concentration and size of extracellular vesicles (EV) isolated from the serum of 3 docetaxel resistant, castration-resistant prostate cancer (CRPC) patients and 3 docetaxel sensitive CRPC patients; The size distribution and relative concentration were calculated by the Nanosight software. **(B)** Protein concentration measurements of the EVs isolated from 3 docetaxel sensitive CRPC patients and 3 docetaxel resistant CRPC patients; **(C)** Western blot analysis of 10 μg of CRPC Tax-Sen and CRPC Tax-Res EVs, probed for MDR-1/3, PABP4 and the exosomal markers Rab5, Alix and CD9.

In order to establish an initial clinical relevance to our data obtained with our cell line model system, we collected serum from 6 castration resistant prostate cancer (CRPC) patients, 3 that are clinically diagnosed with docetaxel resistant CRPC and 3 that are docetaxel sensitive (Table 1). We isolated extracellular vesicles (EV) from the serum and measured by NTA, the relative particle concentration (Figure 5A and Supplementary Figure 3). We found that the median number of particles present in the serum of CRPC Tax-Res patients was higher than those observed in CRPC Tax-Sen ones. We measured the protein concentration of the isolated extracellular vesicles for each patient and it was evident that the resistant patients have higher protein concentration of extracellular vesicles compared to the sensitive ones (Figure 5B). Importantly, we wanted to validate the presence of MDR-1/3 as well as PABP4 in the isolated EVs from the patient serum samples. We observed a good correlation to our cell line data, for MDR-1/3 and PABP4 that could mainly be detected in the EVs isolated from CRPC Tax-Res patients (Figure 5C). The presence of isolated EVs in our processed samples was confirmed by the presence of Rab 5, Alix and CD9 (Figure 5C). In this small pilot study, the *ex vivo* data are consistent with the obtained, *in vitro*, data suggesting that EVs may be used as a source of predictive biomarkers. To validate the predictive significance of these two biomarkers and of other putative biomarkers identified in this study, we will use a larger patients’ cohort.

**Table 1:** Details of patients included in this study

Patient	Clinical stage	Gleason score	Age^(years)^	SerumPSA^#^	Number of Cycles of Docetaxel^##^	Time interval since last dose of docetaxel (months)^&^	de novo resistance	acquired resistance
1	STAGE IV	3 + 4	56	40	8	16	no	yes
2	STAGE IV	4 + 5	75	137	8	3	no	yes
3	STAGE IV	4 + 5	59	64	7	2	no	yes
4	STAGE IV	5 + 4	57	16	9	12	no	no
5	STAGE IV	4 + 4	60	24	7	12	no	no
6	STAGE IV	3 + 4	65	178	10	6	no	no

### Functional properties of docetaxel resistant exosomes

**Figure 6:**
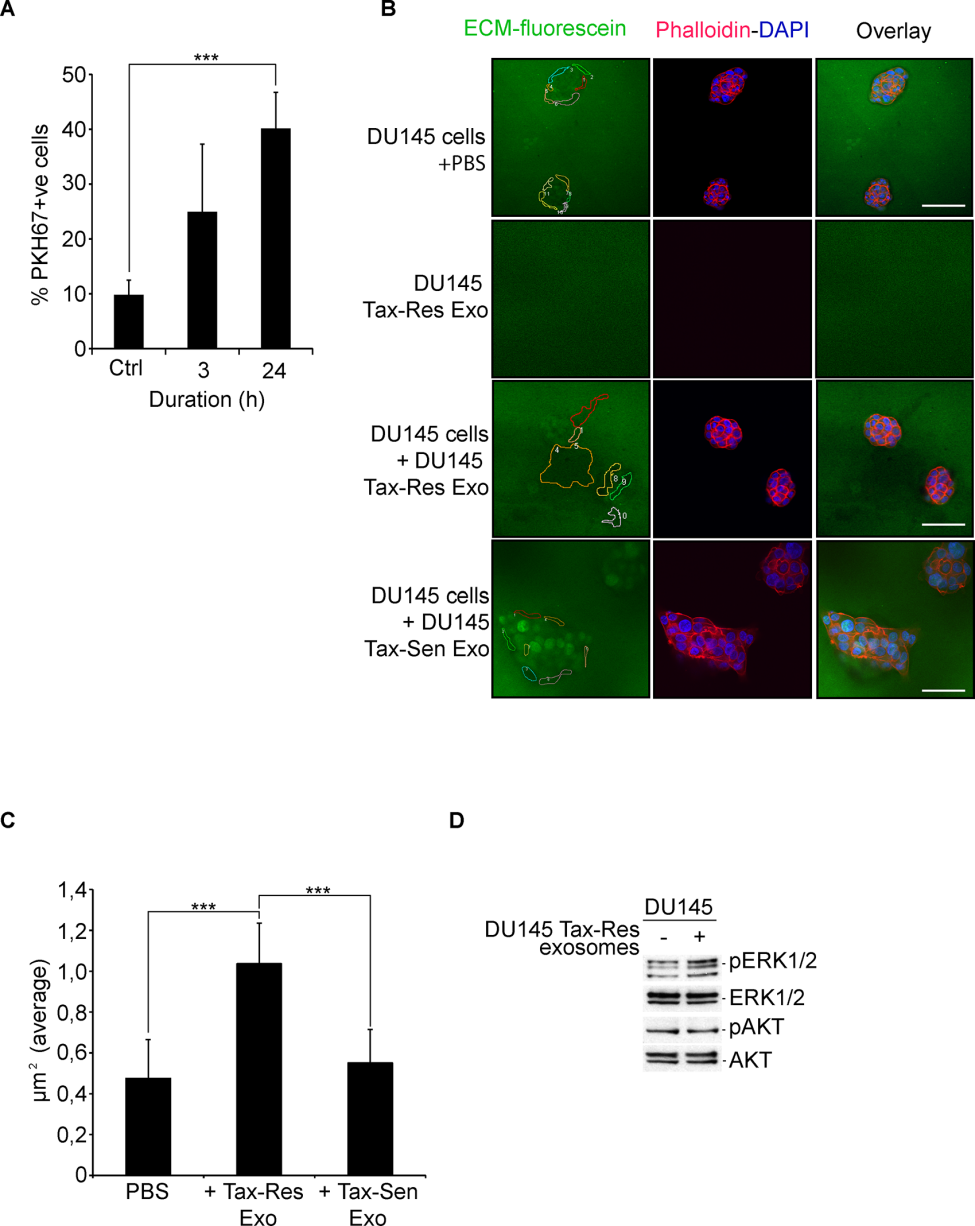
DU145 Tax-Res exosomes promote DU145 cell invasion **(A)** Quantification of PKH67-labeled, DU145 Tax-Res exosome uptake by DU145 cells after 3 hours and 24 hours of incubation. For control, DU145 cells were incubated with PBS for 24 hours (ctrl). The fluorescence intensity was measured by flow cytometry and the percentage of positive cells was measured by the manufacturer's software (means ± SD, *n* = 3, ***P < 0.05); **(B)** Extracellular matrix degradation (ECM) assay on DU145 Tax-Sen cells cultured in the presence of either PBS, 10 μg/ml of DU145 Tax-Res or 10 μg/ml DU145 Tax-Sen cell derived exosomes for 24 hours. DU145 Tax-Res exosomes (10 μg/ml) were cultured together with the ECM-fluorescein for 24 hours. The regions of interest were identified and quantified as described in materials and methods. The DU145 Tax-Sen cells were co-stained with phalloidin for actin cytoskeleton and with DAPI for the nucleus, **(C)** The ECM degradation was quantified as described in materials and methods, (means ± SD, *n* = 3, ***P < 0.05); **(D)** Immunoblot analysis of total and phosphorylated levels of AKT and ERK1/2 in DU145 Tax-Sen cells cultured in the presence or absence of 10 μg/ml of DU145 Tax-Res cell derived exosomes for 24 hours.

We investigated the propensity of DU145 Tax-Res exosomes in transferring the acquired resistance to DU145 Tax-Sen cells. We examined the uptake of DU145 Tax-Res exosomes by DU145 cells in a time-dependent manner after 3 hours and 24 hours (Figure 6A). Incubation of DU145 Tax-Res exosomes with DU145 Tax-Sen cells did not lead to any significant change in the percentage of cell death induced by docetaxel (data not shown). We then examined whether DU145 Tax-Res exosomes could promote the invasive or migratory properties of DU145 Tax-Sen cells [33]. We utilized, a commonly used method, namely the extracellular matrix degradation assay to determine these pro-metastatic properties of DU145 cells that have been educated with DU145 Tax-Res exosomes [34–36]. We found that DU145 cells that have received 10 μg/ml of DU145 Tax-Res exosomes have higher capacity to degrade the extracellular matrix compared to DU145 cells treated with either PBS or DU145 Tax-Sen exosomes (Figure 6B). We also examined whether DU145 Tax-Res exosomes have by themselves an effect on the degradation of the ECM but we could not detect any obvious differences in the fluorescence degradation of the ECM (Figure 6B): The amount of degraded extracellular matrix, depicted as regions of matrix in which fluorescein has been degraded, was quantified and found to be higher in DU145 Tax-Res exosome treated DU145 cells than the matrix of PBS of DU145 Tax-Sen treated DU145 cells (Figure 6C). The Src/PI3K/AKT and the Ras/Raf/MEK/ERK signaling cascades have been shown to be instrumental in promoting migration, invasion and metastasis [37]. We examined whether the addition of DU145 Tax-Res exosomes could modulate the active, phosphorylated levels of AKT and ERK1/2. There was no difference in AKT phosphorylation levels but a modest increase in the levels of ERK1/2 phosphorylation (Figure 6D). Collectively these data indicate that DU145 Tax-Res exosomes may have the propensity to promote migration and invasion in recipient prostate cancer cells. Interestingly, isolated exosomes from PCa patient's sera have been shown to induce enhanced cell proliferation and invasion of the prostate cancer cell lines 22Rv1 and DU145, respectively [22]. The aforementioned data warrant the need for further investigation in clinically relevant mouse models.

To the best of our knowledge, this is the first report describing biochemical and molecular characterization of exosomes secreted by prostate cancer cells that are sensitive or have acquired resistance to docetaxel. We have identified significant differences in the amount of exosomes secreted as well as in their molecular profiles. We have validated our proteomic analysis by western blot and confirmed the presence of some of these biomarkers in serum patient samples. Molecular examination of secreted vesicles from cancer cells may provide a tumor fingerprint that will facilitate the discovery of biology-driven biomarkers that can predict CRPC response to therapy.

## MATERIAL AND METHODS

### Cell lines and culture conditions

DU145 docetaxel resistant cells (DU145 Tax-Res) were generated by growing the cells in 50 ng/ml docetaxel for one week followed by a gradual increase in docetaxel concentration for several weeks up to a concentration of 500 ng/ml (IC50 for the DU145 sensitive cells is 50 ng/ml). DU145 Tax-Sen and DU145 Tax-Res cells were cultured in 75 cm^2^ flasks with RPMI 1640 (Hyclone) enriched with 10% Fetal Bovine Serum (Hyclone), Glutamine (2 mM) Penicillin and Streptomycin (50 μg/ml) (GIBCO). For exosome isolation they were cultured for 48 hours in exosome depleted medium as described below.

### Antibodies and reagents

The primary antibodies used in this study against Rab 5, CD81, CD82, Alix, Emmprin, Caveolin-1 were obtained from Cell Signaling Technology; TSG101, CD9 and CD82 from Abcam, MDR-1 and MDR-3 from Gene Tex; AIF, CD9, Endophilin A2 and CD63 from Santa Cruz Biotechnology; PACSIN2 from Abcam and Santa Cruz Biotechnology; Cyp17A from Novus Biologicals; IgG1-APC from Biolegend. All conjugated antibodies including CD9, CD63, CD81 and CD82 were obtained from BD Pharmingen.

### Patient samples

Whole blood samples (8 ml) for exosome analyses were retrieved from patients with metastatic castration resistant prostate cancer treated in the ongoing prospective clinical trial Concab (EudraCT 2011–004178-27). In this trial, two different regimens of cabazitaxel are compared in a 1:1 randomized design; i.e. cabazitaxel at 25 mg/ml at three week intervals with 10 mg/ml cabazitaxel, given weekly for five of six consecutive weeks. Patients in both treatment groups receive continuous prednisone/prednisolone. Blood samples are collected at baseline before treatment initiation and consecutively, prior to every new treatment cycle. The primary endpoint is to compare the two treatment arms with respect to the total cumulative dose of cabazitaxel received in relation to the planned full dose at the 18 week time interval. All patients included in the trial have previously been treated with docetaxel but stopped treatment due to disease progression (i.e. docetaxel resistant patients) or due to poor tolerability (i.e. docetaxel sensitive patients) which means that samples from both docetaxel resistant and sensitive patients were available for analyses.

### Exosome isolation protocol

To prepare exosome depleted media we centrifuged RPMI 1640 enriched with 30% fetal bovine serum overnight at 120,000 g, 4°C. This exosome depleted medium was further diluted with RPMI 1640 to reach the 10% FBS final concentration which is used for the subsequent culturing of the cells and collection of the supernatants. For isolation of exosomes, we cultured the cells with 70% confluence in multilayer flasks (Millicell® HY flask from Milipore), substituted the culture medium to exosome depleted medium and cultured them for 48 hours. The supernatant was collected and filtered through a 0.22 μm disposable filter. The filtered supernatant was centrifuged for 120 minutes at 120,000 g, 4°C. The pellet was resuspended in PBS and centrifuged again for 120 minutes at 120,000 g, 4°C. The pellet was resuspended in an appropriate volume of PBS and stored in aliquots at −80°C.

For exosome isolation from patient samples, 3 ml serum of each patient were used. The serum was centrifuged at 1500 g for 10 minutes, 4°C and the supernatant was collected and ran again at 12,000 g for 30 minutes, 4°C. The supernatant was collected and filtered through a 0.22 μm disposable filter. The filtered supernatant was centrifuged for 120 min at 120,000 g, 4°C. The pellet was resuspended in PBS and centrifuged again for 120 min at 120,000 g, 4°C. The pellet was resuspended in an appropriate volume of PBS and stored in aliquots at −80°C. Protein content was measured by BCA assay (Thermo Scientific).

### Sucrose density gradient

We made 0.2 to 2 M sucrose gradient as previously described [38]. We placed our exosomal preparation on the surface of the sucrose gradient and ultracentrifuged for 20 hours, 120,000 g at 4°C. The collected fractions were used for further exosomal characterization: i) for western blotting and flow cytometry, the fractions were ultracentrifuged at 120,000 g for 2 hours, 4°C and processed as described in the western blot and flow cytometry methods, respectively; ii) for Nanosight analysis, the sucrose gradient fractions were used directly as described in the NTA method section.

### Nanoparticle tracking analysis (NTA)

We used an NS500 (NanoSight Limited, London, UK) equipped with an 8 mega pixel camera (Andor Technology, Tokyo, Japan) and a 405 nm laser to measure the size and concentration of exosomes in our samples. NTA v2.3.0.17 software (NanoSight Limited) was used for both data acquisition and analysis. We measured three independent samples for each experiment and then we showed their average. Length of each video was 90 seconds with camera level of 14 and detection threshold of 15 during analysis.

### Protein extraction and digestion

The pellets were solubilized in 300 μl of 500 mM ammonium bicarbonate and 1% SDS and sonicated for 1 minute at an amplitude of 20% with 5 pulsed of 5 second (Vibra-Cell™ CV18, Sonics & Materials, Newtown, USA). After acetone precipitation the proteins were resuspended in 0.1% ProteaseMax (Promega), 50 mM ammonium bicarbonate and 10% acetonitrile and protein yields were between 10 and 85 μg. Five μg of each sample were incubated for 30 minutes at 50°C followed by an additional bath soncication of 10 minutes at room temperature. Samples were centrifuged and the supernatant was directly subjected to a tryptic digestion protocol carried out by a liquid handling robot (MultiProbe II, Perkin Elmer). This included protein reduction in 5 mM DTT at 56°C and alkylation in 15 mM iodacetamide for 30 minutes at room temperature in the dark. Trypsin was added in an enzyme to protein ratio of 1:30 and digestion was carried out over night at 37°C. Samples were run in duplicates.

### Liquid chromatography tandem mass spectrometry

Tryptic peptides were cleaned with C18 StageTips (Thermo Fisher Scientific Inc) and 0.5 μg of the resulting peptide mixture was injected into an nano-Ultimate system (Thermo Scientific) in-line coupled to a QExactive mass spectrometer (Thermo Scientific, Bremen, Germany). The chromatographic separation of the peptides was achieved using an in-house packed column (C18-AQ ReproSil-Pur®, Dr. Maisch GmbH, Germany) with the following gradient: 5–35% acetonitrile for 89 minutes, 48–80% ACN for 5 minutes and 80% ACN for 8 minutes all at a flow rate of 300 nl/min. The MS acquisition method was comprised of one survey full scan ranging from m/z 300 to m/z 1650 acquired with a resolution of R = 70,000 at m/z 400, followed by data-dependent HCD scans from maximum ten most intense precursor ions with a charge state ≥ 2. MS2 scans were acquired with a resolution of R = 17,500, a target value of 2e^5^, isolation width was set to 4 and normalized collision energy to 26.

### Data analysis

Tandem mass spectra were extracted using Raw2MGF (in-house developed software), and the resulting mascot generic files were searched against a SwissProt protein database (reversed protein sequences had been added to database for decoy search) using the Mascot 2.3.0 (Matrix Science Ltd.). Mascot was set up to search a concatenated SwissProt protein database (selected for Homo sapiens) using trypsin and allowing for one missed cleavage sites. Peptide mass tolerance was set to 10 ppm and 0.02 Da for the fragment ions. Carbamidomethylation of cysteine was specified as a fixed modification, whereas oxidation of methionine and deamidation of asparagine and glutamine were defined as variable modifications.

Quantitative information was extracted using in-house developed software Quanti [39]. This software performs extracted ion current quantification. For quantitative purposes only peptides identified with a Mascot Score 18 were selected. Such a threshold was set to fulfil condition of no more than 1% of FDR over total peptide population. Only proteins with at least two peptides were considered for quantitation. The hierarchical clustering in Figure 4A was generated in Perseus (MaxQuant 2.4) and was done for row and column tree using the following parameters: euclidean distance, linkage method average and k-means preprocessing. The input data were the most abundant 100 proteins.

### Flow cytometry

To detect exosomes by flow cytometry first we bound exosomes to latex beads (Invitrogen, A37304) and then we stained them with conjugated fluorescent antibodies. Briefly, we washed 10 μl of 4 μm latex beads with 1 ml PBS twice and incubated it with 5 μg of purified exosomes for 30 minutes with gentle agitation, at room temperature. Then PBS was added to a final volume of 1 ml, and incubated on a test tube rotator wheel overnight at 4°C. Then we washed the beads and resuspended in blocking buffer and incubated it for 30 minutes with gentle agitation at room temperature. The beads were then washed once more, divided into several tubes and conjugated antibodies against CD9, CD63, CD81 and CD82 were added. The appropriate isotype controls were used. The beads were incubated for 30 minutes, washed twice and ran on a FACS Calibur flow cytometer (BD Biosciences, California, USA). The data were analyzed and quantified by using the manufacturer's software, CellQuest.

### Cell death measurements

Redistribution of plasma membrane phosphatidylserine is a marker of apoptosis and was assessed by Annexin V fluorescein isothiocyanate (FLUOS) (Roche, 14461000). Briefly, 2 × 10^5^ cells per sample were collected, washed in PBS, pelleted and re-suspended in incubation buffer (10 mM HEPES/NaOH, pH 7.4, 140 mM NaCl, 5 mM CaCl_2_) containing 1% Annexin V and PI. Samples were kept in the dark and incubated for 10 minutes prior to addition of another 400 μl of incubation buffer and subsequent analysis on Calibur flow cytometer (Becton Dickinson, San José, CA, USA) using Cell Quest software.

### Western blotting

Cells were harvested and homogenized in RIPA lysis buffer (10 mM Tris, pH 7.2, 150 mM NaCl, 1% deoxycholate, 1% Triton, 0.1% SDS, 5 mM EDTA) containing complete protease inhibitor cocktail, phospho-stop (Roche Diagnostics, Meylan, France), dithiothreitol (Sigma Aldrich) and vanadate (Life technologies). The exosomes were centrifuged for 120 minutes at 120,000 g, 4°C. The supernatants were discarded and the pellets were lysed by complemented RIPA lysis buffer. After 1 hour on ice, the samples were sonicated and protein quantification was carried out with a Bio-Rad protein assay. Equal amounts of soluble protein (15–25 μg) were denaturated by heating at 95°C for 5 minutes, resolved in sodium dodecyl sulphate-polyacrylamide gel electrophoresis (SDS-PAGE) and transferred to PVDF membranes. The membranes were blocked in 5% non-fat dry milk in TBS-T for 1 hour and probed initially with specific primary antibody and horseradish peroxidase-conjugated secondary antibody. The protein bands were detected by chemiluminescence (Supersignal, Pierce) exposure on X-ray films (Kodak).

### Electron microscopy -negative staining

An aliquot of 3 μl from samples were added to a grid with a carbon supporting film for 5 minutes. The excess solution was soaked off by a filter paper and the grid was rinsed by adding 5 μl distilled water for 10 seconds, soaked off and stained with 1% uranyl acetate in water for 10 seconds and then air-dried. The samples were examined in a Tecnai 12 Spirit Bio TWIN transmission electron microscope (FEI Company, Eindhoven, The Netherlands) at 100 kV. Digital images were captured by using a Veleta camera (Olympus Soft Imaging Solutions, GmbH, Münster, Germany).

### Exosome PKH67-labeling

DU145 docetaxel resistant cell-derived exosomes were labelled with PKH67 green fluorescent (PKH67 Green Fluorescent Cell Linker Midi Kit for General Cell Membrane Labeling, #MIDI67, SIGMA) according to the manufacturer's instructions. Briefly, exosomes were labelled with 2.5 μM of PKH67 dye in 400 μl of diluent C for 5 minutes, then blocked for 1 minute in blocking buffer (1% of bovine serum albumin), after which exosomes were washed with PBS by ultracentrifugation at 120,000 g, for 2 hours, 4°C. PKH67-labeled exosomes were then resuspended in PBS and stored at −80°C.

### Exosome uptake assay

In 8-well chamber slides (Nunc™ Lab-Tek™ II Chamber Slide™ System, Thermoscientific Cat. #154534), DU145 Tax-Sen cells were seeded at a density of 2×10^4^/well, in exosome depleted medium. After 48 hours, the medium was replaced with serum-free medium and the cells were incubated with 10 μg/ml of PKH67 labelled exosomes for 3 and 24 hours. At the end of the incubation period, the cells were harvested, washed 3 times with PBS containing 0,5% BSA and the percentage of green fluorescence intensity was measured by using the BD FACS LSRII flow cytometer. The results were analysed by using the FACSDiva software (BD Biosciences).

### ECM degradation assay

The QCMTM Gelatin Invadopodia Assay (Green) (Millipore, Cat. #ECM670), was used according to the instructions of the manufacturer. Briefly, the chambers of 8-well chamber slides (Nunc™ Lab-Tek™ II Chamber Slide™ System, Thermoscientific Cat. #154534) were coated with 1X Poly-L-Lysine for 20 minutes at first and then fixed with 1X Glutaraldehyde for 15 minutes. Afterwards, the gelatin matrix, containing 1X Fluorescein-Gelatin and 1X Unlabeled Gelatin with 1:5 ratio, was added for 10 minutes in dark. The substrate was disinfected with 70% ethanol for 30 minutes and the chambers were washed with growth media for an additional 30 minutes, protected from light. 2×10^4^ of DU145 cells were seeded per well, in serum free media and the slides were placed in a tissue culture incubator at 37°C, 5% CO_2_, for 24 hours. The DU145 cells were treated with 10 μg/ml of Tax-Sen and Tax-Res exosomes, in triplicates for 24 hours. We have also used 10 μg/ml of DU145 Tax-Res exosomes in chambers that contain only the ECM. Fixation was executed with 3.7% formaldehyde for 30 minutes and the samples were blocked in blocking/permeabilisation buffer, which contained 2% FBS and 0.25% Digitonin diluted in DPBS, for 10 minutes. The fluorescent staining was performed with 1:50 TRITC-phalloidin, 2 μg final concentration, and 1:100 DAPI, 1 μg/ml final concentration, for 1 hour. Slides were visualized on an Olympus FV-1000 Confocal Laser Scanning Microscope. Cellular footprints were calculated using the Olympus FV10-ASW software. In brief, cellular footprints were outlined into ROIs (Regions Of Interest) and their area (μm^2^). Standard deviation and *p* values were calculated by the software. All measurements were done in triplicates for each sample well.

## SUPPLEMENTARY FIGURES AND TABLE





## References

[1] Tannock IF, de Wit R, Berry WR, Horti J, Pluzanska A, Chi KN, Oudard S, Theodore C, James ND, Turesson I, Rosenthal MA, Eisenberger MA, Investigators TAX (2004). Docetaxel plus prednisone or mitoxantrone plus prednisone for advanced prostate cancer. The New England journal of medicine.

[2] Berthold DR, Pond GR, Soban F, de Wit R, Eisenberger M, Tannock IF (2008). Docetaxel plus prednisone or mitoxantrone plus prednisone for advanced prostate cancer: updated survival in the TAX 327 study. Journal of clinical oncology: official journal of the American Society of Clinical Oncology.

[3] Kavallaris M (2010). Microtubules and resistance to tubulin-binding agents. Nature reviews Cancer.

[4] Seruga B, Ocana A, Tannock IF (2011). Drug resistance in metastatic castration-resistant prostate cancer. Nature reviews Clinical oncology.

[5] Prensner JR, Rubin MA, Wei JT, Chinnaiyan AM (2012). Beyond, PSA: the next generation of prostate cancer biomarkers. Science translational medicine.

[6] Lilja H, Ulmert D, Vickers AJ (2008). Prostate-specific antigen and prostate cancer: prediction, detection and monitoring. Nature reviews Cancer.

[7] Gercel-Taylor C, Atay S, Tullis RH, Kesimer M, Taylor DD (2012). Nanoparticle analysis of circulating cell-derived vesicles in ovarian cancer patients. Analytical biochemistry.

[8] Taylor DD, Gercel-Taylor C (2008). MicroRNA signatures of tumor-derived exosomes as diagnostic biomarkers of ovarian cancer. Gynecologic oncology.

[9] Drake RR, Kislinger T (2014). The proteomics of prostate cancer exosomes. Expert review of proteomics.

[10] Duijvesz D, Burnum-Johnson KE, Gritsenko MA, Hoogland AM, Vredenbregt-van den Berg MS, Willemsen R, Luider T, Pasa-Tolic L, Jenster G (2013). Proteomic profiling of exosomes leads to the identification of novel biomarkers for prostate cancer. PloS one.

[11] Dijkstra S, Birker IL, Smit FP, Leyten GH, de Reijke TM, van Oort IM, Mulders PF, Jannink SA, Schalken JA (2014). Prostate cancer biomarker profiles in urinary sediments and exosomes. The Journal of urology.

[12] Llorente A, Skotland T, Sylvanne T, Kauhanen D, Rog T, Orlowski A, Vattulainen I, Ekroos K, Sandvig K (2013). Molecular lipidomics of exosomes released by PC-3 prostate cancer cells. Biochimica et biophysica acta.

[13] Gabriel K, Ingram A, Austin R, Kapoor A, Tang D, Majeed F, Qureshi T, Al-Nedawi K (2013). Regulation of the tumor suppressor PTEN through exosomes: a diagnostic potential for prostate cancer. PloS one.

[14] Hessvik NP, Phuyal S, Brech A, Sandvig K, Llorente A (2012). Profiling of microRNAs in exosomes released from PC-3 prostate cancer cells. Biochimica et biophysica acta.

[15] Duijvesz D, Luider T, Bangma CH, Jenster G (2011). Exosomes as biomarker treasure chests for prostate cancer. European urology.

[16] Tian T, Zhu YL, Hu FH, Wang YY, Huang NP, Xiao ZD (2013). Dynamics of exosome internalization and trafficking. Journal of cellular physiology.

[17] Tian T, Wang Y, Wang H, Zhu Z, Xiao Z (2010). Visualizing of the cellular uptake and intracellular trafficking of exosomes by live-cell microscopy. Journal of cellular biochemistry.

[18] Heijnen HF, Schiel AE, Fijnheer R, Geuze HJ, Sixma JJ (1999). Activated platelets release two types of membrane vesicles: microvesicles by surface shedding and exosomes derived from exocytosis of multivesicular bodies and alpha-granules. Blood.

[19] Bobrie A, Colombo M, Krumeich S, Raposo G, Thery C (2012). Diverse subpopulations of vesicles secreted by different intracellular mechanisms are present in exosome preparations obtained by differential ultracentrifugation. Journal of extracellular vesicles.

[20] Penninger JM, Kroemer G (2003). Mitochondria, AIF and caspases—rivaling for cell death execution. Nature cell biology.

[21] Kjaerulff O, Brodin L, Jung A (2011). The structure and function of endophilin proteins. Cell biochemistry and biophysics.

[22] Corcoran C, Rani S, O'Brien K, O'Neill A, Prencipe M, Sheikh R, Webb G, McDermott R, Watson W, Crown J, O'Driscoll L (2012). Docetaxel-resistance in prostate cancer: evaluating associated phenotypic changes and potential for resistance transfer via exosomes. PloS one.

[23] van Helvoort A, Smith AJ, Sprong H, Fritzsche I, Schinkel AH, Borst P, van Meer G (1996). MDR1 P-glycoprotein is a lipid translocase of broad specificity, while MDR3 P-glycoprotein specifically translocates phosphatidylcholine. Cell.

[24] Ciravolo V, Huber V, Ghedini GC, Venturelli E, Bianchi F, Campiglio M, Morelli D, Villa A, Della Mina P, Menard S, Filipazzi P, Rivoltini L, Tagliabue E, Pupa SM (2012). Potential role of HER2-overexpressing exosomes in countering trastuzumab-based therapy. Journal of cellular physiology.

[25] Derry MC, Yanagiya A, Martineau Y, Sonenberg N (2006). Regulation of poly(A)-binding protein through PABP-interacting proteins. Cold Spring Harbor symposia on quantitative biology.

[26] Valadi H, Ekstrom K, Bossios A, Sjostrand M, Lee JJ, Lotvall JO (2007). Exosome-mediated transfer of mRNAs and microRNAs is a novel mechanism of genetic exchange between cells. Nature cell biology.

[27] Attard G, Reid AH, A'Hern R, Parker C, Oommen NB, Folkerd E, Messiou C, Molife LR, Maier G, Thompson E, Olmos D, Sinha R, Lee G, Dowsett M, Kaye SB, Dearnaley D (2009). Selective inhibition of CYP17 with abiraterone acetate is highly active in the treatment of castration-resistant prostate cancer. Journal of clinical oncology: official journal of the American Society of Clinical Oncology.

[28] Ryan CJ, Smith MR, Fong L, Rosenberg JE, Kantoff P, Raynaud F, Martins V, Lee G, Kheoh T, Kim J, Molina A, Small EJ (2010). Phase I clinical trial of the CYP17 inhibitor abiraterone acetate demonstrating clinical activity in patients with castration-resistant prostate cancer who received prior ketoconazole therapy. Journal of clinical oncology: official journal of the American Society of Clinical Oncology.

[29] Reid AH, Attard G, Danila DC, Oommen NB, Olmos D, Fong PC, Molife LR, Hunt J, Messiou C, Parker C, Dearnaley D, Swennenhuis JF, Terstappen LW, Lee G, Kheoh T, Molina A (2010). Significant and sustained antitumor activity in post-docetaxel, castration-resistant prostate cancer with the CYP17 inhibitor abiraterone acetate. Journal of clinical oncology: official journal of the American Society of Clinical Oncology.

[30] Hao J, Madigan MC, Khatri A, Power CA, Hung TT, Beretov J, Chang L, Xiao W, Cozzi PJ, Graham PH, Kearsley JH, Li Y (2012). *In vitro* and *in vivo* prostate cancer metastasis and chemoresistance can be modulated by expression of either CD44 or CD14. PloS one.

[31] Thompson TC, Tahir SA, Li L, Watanabe M, Naruishi K, Yang G, Kadmon D, Logothetis CJ, Troncoso P, Ren C, Goltsov A, Park S (2010). The role of caveolin-1 in prostate cancer: clinical implications. Prostate cancer and prostatic diseases.

[32] Yang G, Truong LD, Wheeler TM, Thompson TC (1999). Caveolin-1 expression in clinically confined human prostate cancer: a novel prognostic marker. Cancer research.

[33] Kahlert C, Kalluri R (2013). Exosomes in tumor microenvironment influence cancer progression and metastasis. Journal of molecular medicine.

[34] Wang Y, McNiven MA (2012). Invasive matrix degradation at focal adhesions occurs via protease recruitment by a FAK-p130Cas complex. The Journal of cell biology.

[35] Steinestel K, Bruderlein S, Lennerz JK, Steinestel J, Kraft K, Propper C, Meineke V, Moller P (2014). Expression and Y435-phosphorylation of Abelson interactor 1 (Abi1) promotes tumour cell adhesion, extracellular matrix degradation and invasion by colorectal carcinoma cells. Molecular cancer.

[36] Artym VV, Yamada KM, Mueller SC (2009). ECM degradation assays for analyzing local cell invasion. Methods in molecular biology.

[37] Kinkade CW, Castillo-Martin M, Puzio-Kuter A, Yan J, Foster TH, Gao H, Sun Y, Ouyang X, Gerald WL, Cordon-Cardo C, Abate-Shen C (2008). Targeting AKT/mTOR and ERK MAPK signaling inhibits hormone-refractory prostate cancer in a preclinical mouse model. The Journal of clinical investigation.

[38] Thery C, Amigorena S, Raposo G, Clayton A, Bonifacino Juan S (2006). Isolation and characterization of exosomes from cell culture supernatants and biological fluids. Current protocols in cell biology / editorial board.

[39] Lyutvinskiy Y, Yang H, Rutishauser D, Zubarev RA (2013). In silico instrumental response correction improves precision of label-free proteomics and accuracy of proteomics-based predictive models. Molecular & cellular proteomics: MCP.

