# Supplementary material for: Molecular profiling of prostate cancer derived exosomes may reveal a predictive signature for response to docetaxel
Source: Oncotarget. 2015 Mar 12;6(25):21740–54. doi: 10.18632/oncotarget.3226 (PMC4673300; doi:10.18632/oncotarget.3226)
Supplement: Supplementary file 1 [file oncotarget-06-21740-s001.pdf]

SUPPLEMENTARY FIGURES AND TABLE

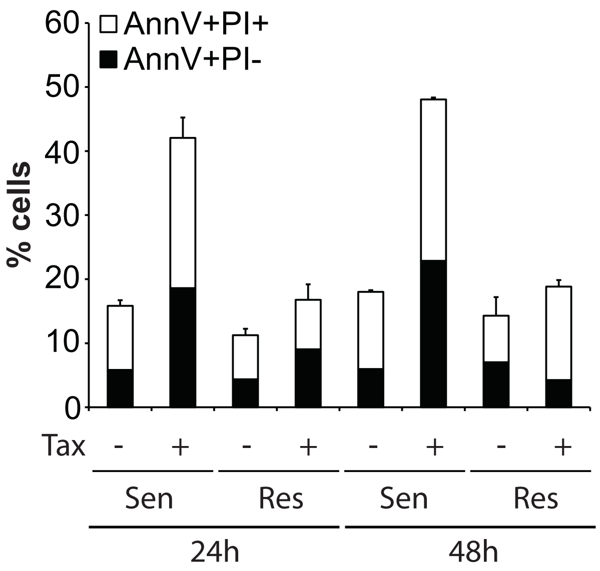

Supplementary Figure 1: DU145 Tax-Res cells do not undergo cell death in response to docetaxel. Quantitative analyses of Annexin V/PI positive DU145 cells treated with 50 ng/ml docetaxel for 24 and 48 hours (means ± SD, *n* = 3).

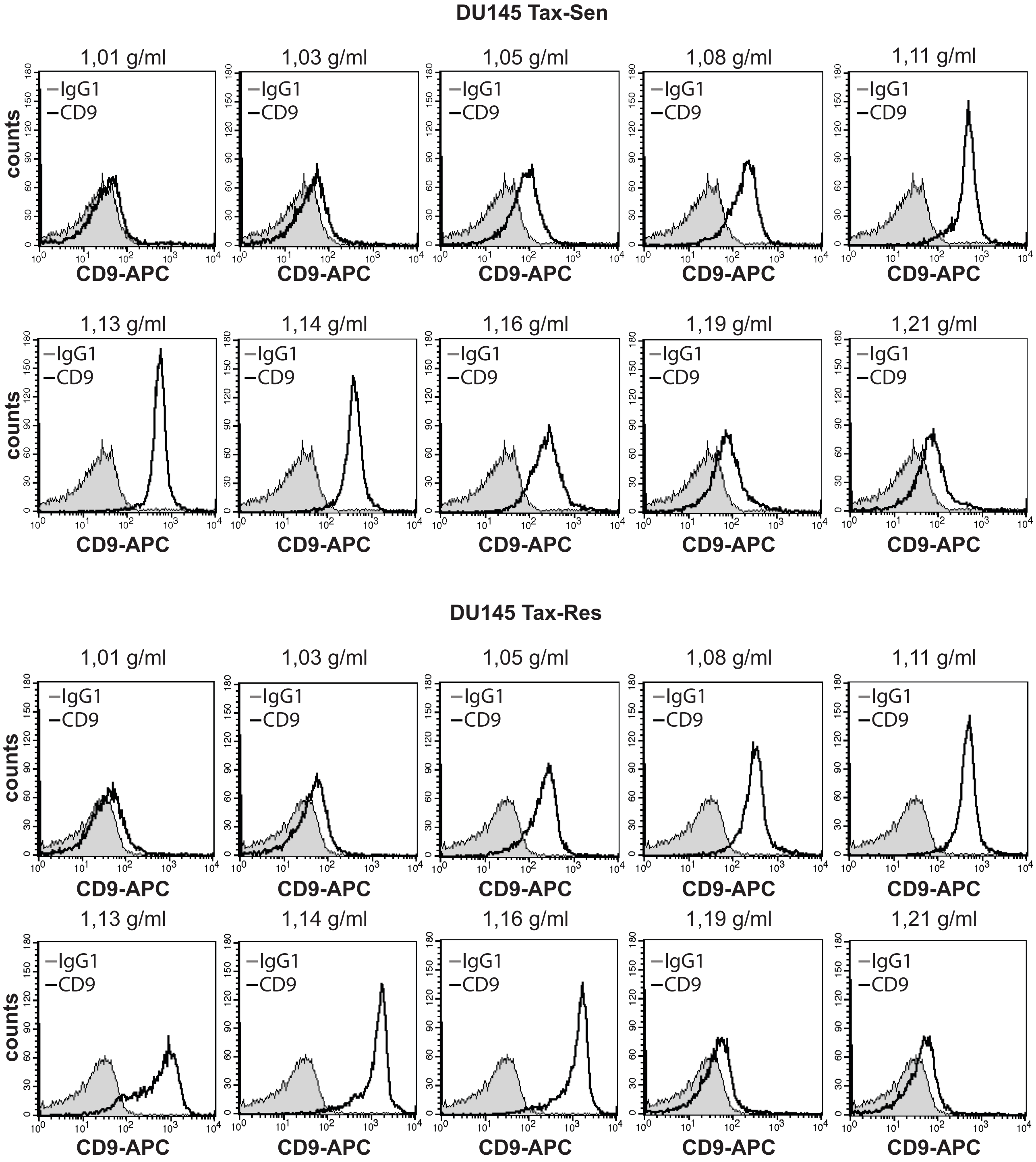

Supplementary Figure 2: Flow cytometric analysis of the sucrose gradient fractions for the DU145 Tax-Sen and Tax-Res exosomes. Flow cytometric analysis of CD9-APC fluorescence intensity in the individual fractions of the sucrose gradient.

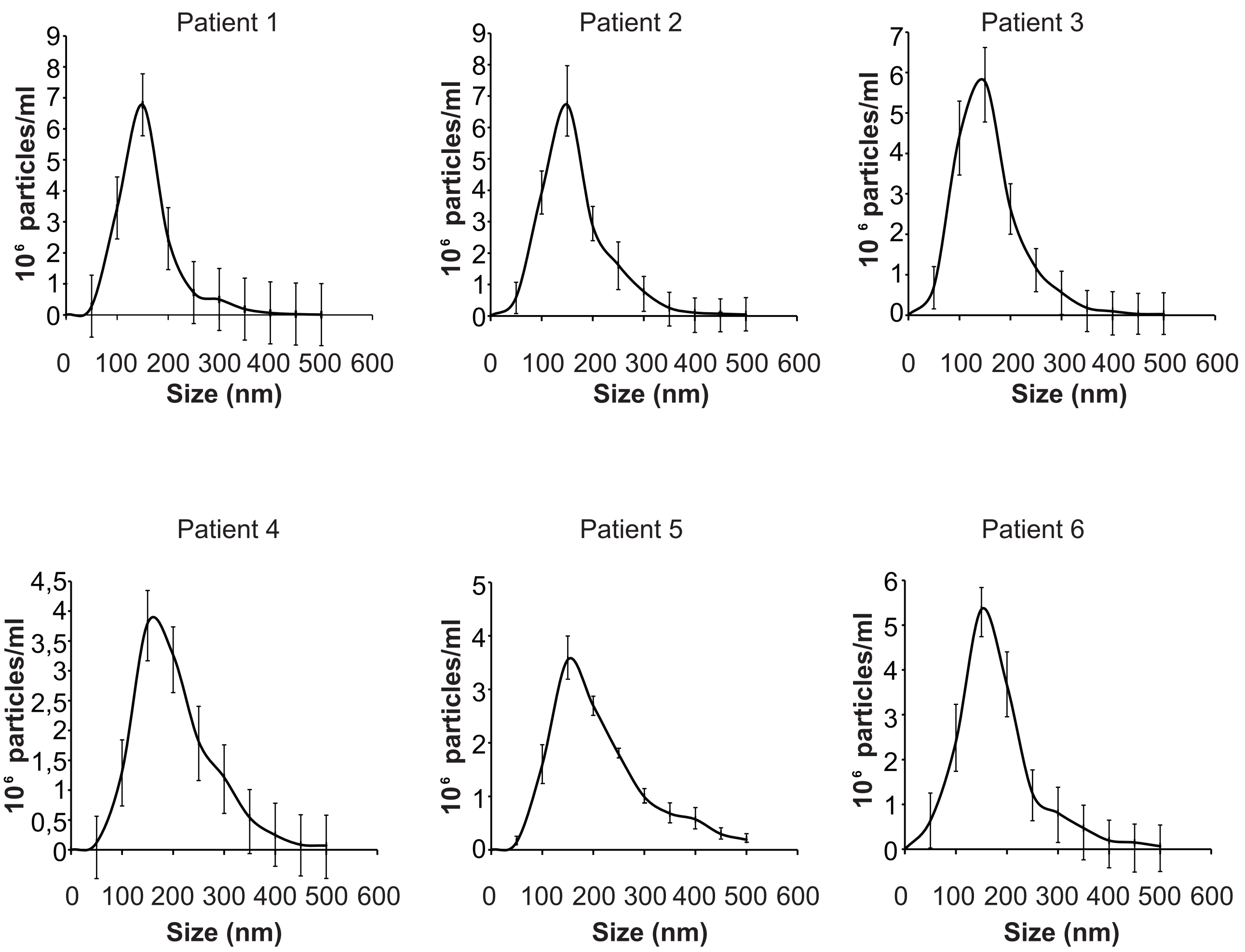

Supplementary Figure 3: Nanoparticle tracking analysis of the CRPC patient samples. Nanoparticle tracking analysis of the relative concentration and size of EVs isolated from the serum of 3 docetaxel resistant and 3 docetaxel sensitive CRPC patients, as described in the materials and methods. The size distribution and relative concentration were calculated by the Nanosight software (means ± SD, *n* = 3).

Supplementary Table 1: List of proteins identified by LC MS/MS proteomics analysis of DU145 Tax-Sen and DU145 Tax-Res derived exosomes
